# Preference for cesarean section in young nulligravid women in eight OECD countries and implications for reproductive health education

**DOI:** 10.1186/s12978-017-0354-x

**Published:** 2017-09-12

**Authors:** Kathrin H. Stoll, Yvonne L. Hauck, Soo Downe, Deborah Payne, Wendy A. Hall, International Childbirth Attitudes- Prior to Pregnancy (ICAPP) Study Team, Mechthild Gross, Michelle Sadler, Gillian Thomson, Joana Steffing, Anne Malott, Patricia McNiven, Judith McAra-Couper, Emma Swift, Joyce Edmonds

**Affiliations:** 1 School of Population & Public Health & Division of Midwifery, University of British Columbia, 2206 E Mall, Vancouver, BC V6T 1Z9 Canada; 2 School of Nursing, Midwifery and Paramedicine, Curtin University and King Edward Memorial Hospital, Perth, WA Australia; 3 School of Community Health and Midwifery, University of Central Lancashire, Preston, UK; 4 Centre for Midwifery & Women’s Health Research & Disability, Diversity & Gender Cluster, PCRC, School of Clinical Sciences, Auckland University of Technology, Auckland, New Zealand; 5 School of Nursing, University of British Columbia, Vancouver, BC Canada

**Keywords:** Cesarean, Women, Fear, Knowledge, Learning needs, Survey

## Abstract

**Background:**

Efforts to reduce unnecessary Cesarean sections (CS) in high and middle income countries have focused on changing hospital cultures and policies, care provider attitudes and behaviors, and increasing women’s knowledge about the benefits of vaginal birth. These strategies have been largely ineffective. Despite evidence that women have well-developed preferences for mode of delivery prior to conceiving their first child, few studies and no interventions have targeted the next generation of maternity care consumers. The objectives of the study were to identify how many women prefer Cesarean section in a hypothetical healthy pregnancy, why they prefer CS and whether women report knowledge gaps about pregnancy and childbirth that can inform educational interventions.

**Methods:**

Data was collected via an online survey at colleges and universities in 8 OECD countries (Australia, Canada, Chile, England, Germany, Iceland, New Zealand, United States) in 2014/2015. Childless young men and women between 18 and 40 years of age who planned to have at least one child in the future were eligible to participate. The current analysis is focused on the attitudes of women (*n* = 3616); rates of CS preference across countries are compared, using a standardized cohort of women aged 18–25 years, who were born in the survey country and did not study health sciences (*n* = 1390).

**Results:**

One in ten young women in our study preferred CS, ranging from 7.6% in Iceland to 18.4% in Australia. Fear of uncontrollable labor pain and fear of physical damage were primary reasons for preferring a CS. Both fear of childbirth and preferences for CS declined as the level of confidence in women’s knowledge of pregnancy and birth increased.

**Conclusion:**

Education sessions delivered online, through social media, and face-to-face using drama and stories told by peers (young women who have recently had babies) or celebrities could be designed to maximize young women’s capacity to understand the physiology of labor and birth, and the range of methods available to support them in coping with labor pain and to minimize invasive procedures, therefore reducing fear of pain, bodily damage, and loss of control. The most efficacious designs and content for such education for young women and girls remains to be tested in future studies.

## Plain English Summary

Cesarean section rates in most high and middle-income countries are higher than recommended and continue to increase. Many strategies have been tested, to reduce rates of Cesarean sections, such as educating clinicians and patients about the benefits of vaginal birth, and the risks of unnecessary Cesarean sections, and ensuring that physicians seek a second opinion before proceeding with surgery. Most of these strategies are not linked to sizable reductions in Cesarean section rates.

In this paper we argue that interventions aimed at reducing the Cesarean section rate should begin before women and men become parents, because attitudes towards birth are developed in advance of pregnancy and might be influenced by modifiable factors such as childbirth fear and lack of knowledge of pregnancy and birth. We studied young women from 8 OECD countries, to better understand how many would prefer a Cesarean section in a healthy pregnancy, why and whether preferences vary across countries. We found that 1 in 10 women would prefer CS, ranging from 7.6% in Iceland to 18.4% in Australia. Fear of labour pain and fear of physical damage were the most common reasons why young women prefer CS. In addition to childbirth fear, young women who preferred CS reported several knowledge gaps and misperceptions about childbirth that can be addressed through education. While findings from the current study can inform educational programming, the best way to deliver education about pregnancy and birth to young women (and men) remains to be tested.

## Background

Globally, an estimated 6.2 million unnecessary cesarean sections (CS) are performed each year, at an approximate cost of 2.3 billion US dollars [1]. Data from 194 countries indicates that a CS rate above 19% is associated with higher maternal and neonatal mortality [2]. In another study of 159 countries, no decreases in maternal or infant mortality were observed with CS rates above 10% [3]. These findings indicate that a CS rate between 10 and 19% is optimal; however, all OECD countries exceed the lower limit of this range, and almost all exceed the higher limit [4].

Differences in CS rates across countries have been attributed to a range of factors, including case mix, financial incentives, fear of malpractice litigation, differences in the availability and training of midwives and nurses, access to out-of-hospital birth options, and the proportion of women who access private maternity care [5–7]. Many strategies have been tested to reduce the number of unnecessary cesarean sections. These include active management of labor [8] continuous labor support [9], mode of delivery decision-aids and information for pregnant women [10, 11] and mandatory second opinions [12]. These and other strategies have been largely ineffective [8, 13].

Recently, psychological indications for cesarean section have gained recognition. In particular, fear of childbirth is linked to a preference for CS during pregnancy and/or giving birth via CS, even in the absence of medical indications [14–17]. For example, Ryding et al. [18] surveyed over 6000 childbearing women across 6 European countries; they found that 16.7% of first time mothers and 31.7% of multiparas with severe fear of childbirth had a CS without medical indications (compared to 4.6% and 17.5% of women without severe fear of birth). The link between childbirth fear and preferences for CS over vaginal births has also been observed among young women from Canada and the United States (US) who plan to become pregnant [19, 20].

Given the iatrogenic morbidities and increased cost associated with unnecessary CS [1], the limited effectiveness of strategies aimed at care providers, pregnant women and institutional structures, and evidence of well-developed birth preferences expressed by young women prior to pregnancy, the objectives of this study were to examine 1) preferences for Cesarean section in a hypothetical healthy pregnancy among young women from 8 OECD countries, 2) reasons for this preference and 3) knowledge gaps and misperceptions about pregnancy and birth among young women that can inform educational strategies.

## Methods

We recruited childless women and men between the ages of 18–40 years from different OECD countries. Data were collected via online survey at ten universities and colleges in eight countries between 2014 and 2015. At each institution, an invitation to the survey was either sent to all students at the university or a subsample of students. For example, at Curtin University in Western Australia the survey invitation was sent to 8000 domestic students, which constitutes 15% of the total student body. At the University of Iceland the invitation was sent to all enrolled students (*N* = 9805). In Germany, two universities participated. At Hannover Medical School all 3130 students were invited and at the University of Bamberg all 12,800 students received the invitation. Students were directed to the consent form once they clicked on the survey link. The consent form described the purpose of study, how anonymity would be preserved and the consent process, i.e. by starting the survey students consented to participate in the study. Ethics approval for the study was granted by the Behavioral Research Ethics Board at the University of British Columbia, Canada (H14–00033) and by institutional review boards at all participating universities and colleges, with the exception of the University of Northern British Columbia (UNBC). Data collection at UNBC was covered by the original ethics approval.

Students completed an online questionnaire with 5 sections: 1) Socio-demographic questions, 2) birth preferences and reasons for preferences, 3) attitudes towards birth, 4) vicarious experiences with childbirth and sources of information that shaped students’ attitudes towards pregnancy and birth, 4) psychological profile (depression, anxiety, stress and childbirth fear) and 5) learning needs/ knowledge gaps about pregnancy and childbirth (see Table 1 for sample items). Depression, anxiety and stress were measured with the 21-item short form of the DASS scale (7 items per construct) [21]. Internal consistency reliability of the DASS-21 ranged from 0.91 among Canadian women in our study to 0.95 among women from the UK. Subscale alphas ranged from 0.83–0.89 for the stress subscale, 0.68–0.83 for the anxiety subscale and 0.87–0.92 for the depression subscale. Fear of childbirth was measured with a 10-item scale that was developed for the cross-country study, the *Childbirth Fear Prior to Pregnancy Scale*. The scale assesses childbirth fear along three domains: 1) Fear of pain and being out of control (5 items), 2) fear of complications (3 items) and 3) fear of physical damage (2 items). The six response options ranged from strongly disagree to strongly agree, with higher scores indicating increased fear. Internal consistency reliability of the scale ranged from 0.85 among women in the US to 0.89 among women from NZ and Iceland. Subscale alphas ranged from 0.83–0.87 for subscale 1, 0.74–0.83 for subscale 2 and 0.89–0.94 for subscale 3. The total scale scores were highly correlated with an established measure of childbirth fear across samples, supporting the construct validity/ convergent validity of the scale. Details about the recruitment, forward backward translation of surveys, scale construction and psychometric testing of the DASS-21 and *Childbirth Fear Prior to Pregnancy (CFPP)* scale are described elsewhere [22].

**Table 1:** Survey sections and sample items

Survey section	Sample items
Socio-demographic profile: age, field of study, country of origin	Were you born in (survey country)?
Birth preferences and reasons for preferences: Preferred mode of delivery Preferred prenatal care provider Preferred place of birth	Assuming the pregnancy is low-risk and you could choose the type of birth for your baby, would you prefer it to be: a vaginal birth or a cesarean birth, i.e., a surgical birth of an infant through an incision in the mother’s abdomen and uterus?
Attitudes towards birth: Attitudes towards obstetric technology and interventions, students’ level of confidence in knowledge of pregnancy and birth	I believe it is a woman’s right to have a Cesarean birth, even if there are no medical indications. I feel confident about my level of knowledge around pregnancy and birth
Experiences with childbirth and sources of information that shaped students’ attitudes towards pregnancy and birth	Have you ever been present for a real (human birth)? Do you feel that your attitudes towards pregnancy/birth were/are shaped by (tick all that apply): visual media, written media, family, friends, school, other.
Psychological profile: DASS-21: Depression, Anxiety, Stress CFPP scale: Childbirth fear	I felt scared without any good reason. I tended to over-react to situations. I am fearful of birth. I feel that I will not be able to handle the pain of childbirth. I am afraid that my body will never be the same again after birth. I fear complications during labor and birth
Learning needs	Please tell us what topics you would be most interested in learning about (tick all that apply): See Table 5 for a list of response options.

Students were asked if they would prefer a vaginal birth or Cesarean birth, assuming the pregnancy is low-risk and they could choose the type of birth for their baby. After students marked their preference for either a Cesarean birth or vaginal birth, they were directed to a list of reasons for their choice. These pre-defined response options were based on a thematic analysis of open-ended comments about mode of delivery preferences of 3680 Canadian students who completed the first version of the survey in 2006 [23].

We report rates of CS preference for all women who responded to the survey and met the eligibility criteria, i.e., they were 40 years of age or younger, not pregnant at the time of data collection, and expressed a desire to have one or more children in the future. Because of very heterogeneous response rates from men (ranging from 35 who responded from the UK to 288 from Chile) we elected to focus our analysis on women only. Further, to account for differences in the age distribution, the number of health sciences students at different universities, and the number of students born outside the survey country, we also report CS rates for a standardized cohort of women aged 18–25, who were born in the survey country, and not enrolled in a health sciences program. To determine whether rates of CS preferences were linked to country level rates we used Spearman’s rho correlational coefficient (r_s_).

To examine whether childbirth fear was associated with preferences for CS, we entered the three childbirth fear domains of the CFPP scale in a logistic regression model, with CS preference as the outcome (reference category: preference for vaginal birth). We controlled for differences in the socio-demographic (age, field of study, country of origin) and psychological profile of students (scores on the Depression Anxiety Stress-21 subscales). We performed this analysis for the full sample and for each country separately, to determine whether results were replicable across samples.

To determine whether childbirth fear and CS preferences in our population might be associated with women’s confidence in their knowledge of pregnancy and birth, we examined CFPP scores and CS preferences for students who reported different levels of agreement with the statement: ‘I feel confident in my level of knowledge of pregnancy and birth’. The six response options for this item ranged from strongly disagree to strongly agree. Finally, for young women with CS preferences and elevated fear of childbirth (i.e. scores above the 75th percentile), we identified pregnancy and childbirth topics that students wanted to learn more about. *P* values are presented, to identify significant differences in knowledge gaps/learning needs for women who were fearful of birth and those who preferred a CS.

## Results

A total of 6571 students started the survey. Response rates in countries where it was known how many students received the invitation to participate were as follows: Australia: 13.2%; Germany: 8.2%; Iceland: 12.0% and USA: 13.5%. A total of 4569 students started the survey, met eligibility criteria and answered the ‘mode of delivery preference’ question. After excluding 942 men who responded to the survey and 11 who did not provide data or preferred not to state their gender, the final sample size for this analysis of female respondents was 3616: 562 responses from Australia (15.5%), 202 from Canada (5.6%), 377 from the USA (10.4%), 313 from the United Kingdom (8.7%), 850 from Germany (23.5%), 478 from Iceland (13.2%), 484 from Chile (13.4%) and 350 from New Zealand (9.7%). Age, field of study, and country of origin differed significantly across countries (*p* < 0.001 for all comparisons).

Overall, 10.8% of study participants expressed a preference for CS in a healthy future pregnancy, ranging from 8.9% of students from in Canada to 16.0% in Australia. When restricting our analysis to the standardized cohort of 18–25 year-old women who were born in the survey country and did not study health sciences (*n* = 1390), we found that proportions of women expressing a preference for CS were still highest in Australia (18.4%). Proportions were lowest in Iceland (7.6%) (see Table 2). Proportions of young women in our study preferring CS were significantly higher in countries with higher national CS rates (r_s_ = 0.04, *p* = 0.03); however, this association was weak and no longer significant when restricting the analysis to the standardized cohort (r_s_ = 0.01; *p* = 0.67).

**Table 2:** Proportion of women who prefer CS in a low risk pregnancy and national CS rates

	N	All	Australia	NZ	UK	USA	Canada	Chile	Germany	Iceland
Preferences for CS in low risk pregnancy- all women	3616	10.8	16.0	10.3	10.2	10.1	8.9	11.8	9.1	9.2
Preferences for CS in low risk pregnancy- standardized cohort^a^	1390	11.7	18.4	14.4	11.9	10.0	14.7	12.8	10.1	7.6
National CS rate^b^	NA	NA	32.4	25.9	26.2	32.2	27.3	56.0	32.9	15.5

^a^women aged 18–25 who do not study health sciences and were born in the survey country

^b^OECD 2013 data for all countries except Chile; Chilean data is from Instituto Nacional de Derechos Humanos, Chile. Situación de los Derechos Humanos en Chile, Informe Anual 2016

The most common reasons expressed by young women for preferring a CS in a healthy future pregnancy were fear of labor pain and avoiding damage to the body/ to maintain vaginal integrity (see Table 3). These reasons were reported by 77.8% and 62.5% of young women who preferred a CS. One in four also reported the ability to plan the time of birth and the convenience of a scheduled CS as reasons for their preferences. A smaller proportion of women (18.1%) expressed a preference for a CS because they believe CS is better/safer and/or healthier for the mother.

**Table 3:** Reasons for CS preference among women from 8 countries (*n* = 392)

Please indicate why you prefer Cesarean birth (CB)	n (%)
Fear of labor pain	305 (77.8)
To avoid damage to my body/to maintain vaginal integrity	245 (62.5)
Ability to plan the time of birth	103 (26.3)
Convenience of scheduled Cesarean birth	102 (26.0)
Cesarean birth is better/safer/healthier for the mother	71 (18.1)
Other	20 (5.1)

Results of the logistic regression analysis across the whole sample of women indicated that health sciences students had significantly lower odds of preferring CS and students with higher scores on the childbirth fear subdomains that measure fear of physical changes and fear of pain/fear of being out of control had significantly increased odds of preferring CS (see Table 4). When performing the same regression analysis for each country separately, we found that fear of complications was not significantly linked to CS preferences in any of the countries. Fear of pain/being out of control was significantly linked to preferences for CS in a healthy future pregnancy in 5 countries (Australia, NZ, the UK, Germany, and Iceland), controlling for differences in students’ socio-demographic and psychological profiles. Fear of physical damage was significantly higher among students who preferred CS in 5 countries (Australia, Canada, Chile, Germany, and Iceland).

**Table 4:** Association between childbirth fear domains and CS preferences, controlling for socio-demographic and psychological profile (*n* = 2988)

	B	Standard error	OR	95% CI
Socio-demographic profile				
Age	0.03	0.02	1.03	1.00–1.06
Born in survey country: Yes (Ref: No)	0.13	0.17	1.14	0.82–1.58
Health sciences student: Yes (Ref: No)	−0.33	0.14	0.72	0.55–0.95
Psychological profile				
Depression	0.02	0.02	1.02	0.98–1.06
Anxiety	0.02	0.03	1.02	0.97–1.07
Stress	−0.01	0.02	0.99	0.95–1.03
Childbirth fear profile				
Fear of complications	−0.02	0.02	0.98	0.94–1.03
Fear of physical changes	0.17	0.03	1.19	1.12–1.25
Fear of pain and being out of control	0.12	0.02	1.12	1.09–1.16

**Fig. 1:**
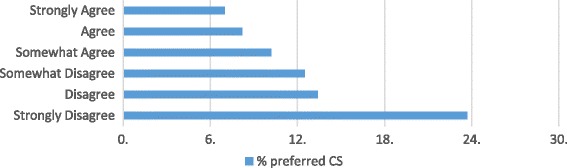
CS preferences, stratified by level of agreement with statement: I feel confident in my level of knowledge of pregnancy and birth (*n* = 3389)

**Fig. 2:**
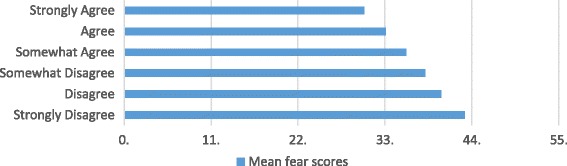
Childbirth fear scores, stratified by level of agreement with statement: I feel confident in my level of knowledge of pregnancy and birth (*n* = 3360)

We found a dose–response relationship between childbirth fear scores and CS preferences: 3.3% of students who scored in the 0-24th percentile on the CFPP scale preferred a CS, 5.1% who scored in the 25th to 49th percentile, 11.3% who scored in the 50th to 74th percentile and 22.9% of students scoring in the top quartile preferred a CS. We also detected a dose- response relationship between confidence in students’ level of knowledge about pregnancy and birth and childbirth fear scores and CS preferences (see Figs. 1 and 2). As confidence in knowledge increased, preferences for CS and childbirth fear decreased.

When asked whether students would like to learn more about pregnancy and childbirth, most said yes (71.1%) or ‘I don’t know’ (13.2%). The topics that most of the young women wanted to learn about were: promotion of a healthy pregnancy (88.0%); the process of labor and birth (84.4%); risks and benefits of common interventions and technologies used during pregnancy, labor and birth (82.8%); the process of pregnancy (81.0%), and what could go wrong during pregnancy, labor and birth (74.4%). Young women with elevated childbirth fear were significantly more likely to identify all topics as important, with the exception of learning about how to promote a healthy pregnancy (see Table 5). A higher proportion of students who expressed a preference for CS in the context of a healthy future pregnancy reported interest in learning about what could go wrong during pregnancy, labor, and birth, compared to students who preferred a vaginal birth (see Table 5). Significantly lower proportions were also interested in learning about how to include their partners in the childbirth experience or learning about out-of-hospital birth options.

**Table 5:** Learning needs of young women who plan to become pregnant, reported for full sample, students who scored above the 75th percentile on the CFPP scale and students who prefer CS

	Full sample %	CFPP scores >75th percentile %	CFPP scores ≤75th percentile %	*p*	Preference for CS %	Preference for vaginal birth %	*P*
The process of pregnancy	81.0	86.9	82.4	0.02	82.6	80.8	0.53
Promotion of a healthy pregnancy (nutrition, life style factors etc.)	88.0	91.6	89.9	0.23	84.7	88.3	0.11
The process of labor and birth	84.4	90.4	85.4	0.002	84.3	84.4	0.97
Available reproductive health services	59.1	69.2	58.6	<0.001	58.3	59.2	0.82
What could go wrong during pregnancy, labor and birth	74.4	85.6	73.4	<0.001	80.6	73.7	0.02
How to include both partners in the childbirth experience	72.0	76.4	73.6	0.22	64.5	72.7	0.02
The anatomy and physiology of the female reproductive system	43.7	56.8	41.6	<0.001	45.9	43.4	0.55
Risks and benefits of common interventions and technologies used during pregnancy, labor and birth	82.8	88.9	83.7	0.002	83.0	82.8	0.95
How the female body is equipped for childbirth	64.7	76.7	63.4	<0.001	66.4	64.5	0.60
Birth at home or at birthing centres	55.8	58.5	57.6	0.73	39.7	57.2	<0.001

## Discussion

The overall rate of CS preference in the context of a healthy pregnancy was 10.8%; this finding is congruent with the proportion of nulliparous women around the world who prefer a CS during pregnancy [24] but is much higher than global estimates of CS on maternal request [25]. In 2013 the CS rate for the countries that were included in this study ranged from 15.5% in Iceland to 56% in Chile [4, 26]. In our study we found that Icelandic students were least likely to express a preferences for CS whereas Australian students had the highest rate of CS preference (standardized cohort), with a difference of over 10%. A brief description of the maternity care systems in Iceland and Australia illustrates potential reasons for these differences. In Iceland, the health care system is publicly funded, and almost all women receive prenatal and intrapartum care from midwives [27]. Icelandic midwives are autonomous providers who are trained to support physiologic labor and birth, and they offer eligible women the option to give birth at home. Just over 2% of babies in Iceland are born at home – which is the highest rate in the Scandinavian countries [28]. These figures suggest a specific cultural bias towards physiological labor and birth.

The Australian sample included women from the state of Western Australia (WA), where, in 2013, 98.4% of women had a hospital birth [29]. Healthcare in Australia involves a two- tiered system of services with public and private sector hospitals. In WA, choices for maternity care include private obstetric care, public hospital care, and midwifery continuity of care through group practices or homebirth with a privately practicing midwife or a publically funded program. WA had the highest (40.3%) proportion of private hospital births in Australia in 2013 [30] and the highest CS rate (34.3%) compared to all states and territories [29]. An increase in pre-labor CS for WA women attending private hospitals has been attributed to the increase in CS rates for nulliparous women [31].

The relatively low proportion of Chilean participants expressing CS preferences compared to actual CS rates contradict assumptions that birth preferences among young women would mimic rates of obstetric interventions at the country level. A study of birth preferences of 180 Chilean women attending public and private antenatal clinics in Santiago showed that 9.4% preferred CS [32]. The authors concluded that Chilean women’s preferences are not a significant contributor to the high rates of CS in the country. The preferences of young Chilean women contemplating pregnancy and birth in our study concur with their conclusion.

We found two main factors that were linked to CS preferences among young women who contemplate pregnancy: fear of uncontrollable pain and fear of physical damage. Epidural analgesia (EA) is very effective at relieving labor pain [33] and might seem like an obvious solution for women with fear of pain. However, evidence from a systematic review of trials comparing EA with other pain relief options or no pain relief during labor showed an increased risk of instrumental vaginal birth, maternal hypotension, and cesarean section for fetal distress for women who received EA. No significant differences in maternal satisfaction with pain relief were noted between the two groups [33]. These findings draw into question EA as a solution to childbirth fear in general and fear of pain in particular, especially when considering that women who experienced an emergency CS or instrumental birth are significantly more likely to rate the experience as negative or traumatic compared to women who had a non-instrumental vaginal birth [34]. Women with a previous negative or upsetting birth experience are significantly more likely to experience fear of birth in a subsequent pregnancy [17, 35, 36]. In other words, while the promise of EA might reduce anticipatory fear of labor pain, EA is not associated with increased satisfaction with pain relief and is linked to interventions that might increase childbirth fear in the longer term.

The link between fear of physical damage and CS preferences has been documented for US, Israeli, and Canadian students who contemplate pregnancy [19, 20, 37]. For example, college students from the US (*n* = 752) who preferred CS were significantly more likely to express elevated concerns about body changes following childbirth, compared to students who preferred a vaginal birth [19]. Similarly, fear of body changes and a preference for CS to prevent physical damage were significantly associated with childbirth fear among Israeli women who had never given birth [37].

Minimal work has examined women’s fears of being out of control during labor and birth. However, some research suggests that this fear might be embedded in internalized gender norms and constructions of vaginal birth as messy and uncontrollable. Martin [38] conducted in-depth interviews with 26 women in the United States within 3 months of giving birth. She found that women worried about being kind, polite, nice, and selfless during labor and birth. These internalized gender norms seemed to exert external control over women and their bodies during childbirth. In a qualitative study with 33 women and 9 maternity care providers from New Zealand, CS was constructed as a routine procedure that is less messy than vaginal birth. Some respondents felt that birth was more controlled, sterile, clean, and contained when having a CS and less embarrassing than a vaginal birth [39]. These findings concur with the results from the current study that show that fear of pain and being out of control strongly correlate with students’ CS preferences.

### A need for education

In many countries, midwives and public health nurses provide preconception care and education to women and men prior to pregnancy. In some countries, like Germany, education about childbirth can start as early as age 8. In Germany, 4 hours of midwifery-led instruction about midwifery care, pregnancy, birth, and newborn care was well received by students in grades 3 and 4 and associated with increased knowledge of pregnancy and birth and decreased childbirth worries [40]. We argue that education about childbirth should be delivered to the next generation of maternity care consumers, commencing as early as primary and secondary school. College and university students would benefit from educational content that addresses fear of physical damage and fear of pain, and all of the topics listed in Table 5. Specifically, young women need to know that a range of effective pain relief options are available to them, and that their bodies will recover faster from a vaginal birth, that mode of delivery is not linked to decreases in sexual functioning or enjoyment [41] and that exercise during the postpartum period can strengthen pelvic floor and core muscles. The content could be presented in workshop format and facilitated by midwifery or obstetric/family practice trainees.

One in five young women who preferred CS for a future pregnancy and birth believed that it is healthier, better, and/or safer for the mother compared to vaginal birth and 83% of students wanted to learn more about the advantages and disadvantages of common obstetric interventions. These findings indicate that students would benefit from a better understanding of the positive outcomes of vaginal birth compared to CS, such as faster recovery time [42], decreased risk of placental disorders in future pregnancies [43], decreased risk of severe maternal morbidity and anesthetic complications [44, 45], and decreased risk of readmission to hospital (for wound complications and infection) [46], as well as health benefits for infants, such as reduced likelihood of developing chronic diseases like asthma or obesity during childhood [47]. Women who have a vaginal birth are also more likely to hold their infants immediately after the birth and have skin-to-skin contact with their newborns, and are significantly more likely to breastfeed at 3 and 6 months compared to mothers who had a CS [48].

Women who preferred CS for a future pregnancy did not report many learning gaps, but were significantly more likely to want to know more about what can go wrong during pregnancy, labour, and birth. When educating young women about birth, it is important to emphasize the overall low risk of serious adverse outcomes and to frame this information in terms of the high likelihood of having a healthy pregnancy and normal birth because the way clinical information is presented can affect risk perception and health care decision-making [49].

Education sessions delivered online, through social media, and face-to-face using drama and stories told by peers (young women who have recently had babies) or celebrities increase young women’s capacities to understand the physiology of labor and birth, and the range of methods available to support them in coping with labor pain and minimizing invasive procedures. Such sessions could potentially reduce fear of pain, bodily damage, and loss of control. The most efficacious designs and content for education for young women and girls requires testing in future studies. The learning needs and knowledge gaps of young men should be explored in future studies, using a larger and more representative sample. Research with Swedish couples indicated that the attitudes of men (specifically prenatal childbirth fear) were strongly linked to decisions about mode of delivery [50], and it is important to include men in any future studies that test educational interventions.

### Limitations

Data presented are based on convenience samples from university students from eight countries; response rates were low, and do not reflect population sizes. For these reasons findings cannot be generalized to all young women from the countries that were included in our study. Nonetheless, we were able to replicate key findings across countries, demonstrate dose–response relationships and use a standardized cohort for cross-country comparisons, to minimize bias. In this study we assessed young women’s confidence about their knowledge of pregnancy and birth. While increased confidence was linked to decreased fear and preferences for CS it is unclear whether confident students actually had more accurate information about pregnancy and birth.

## Conclusions

Young women who contemplate pregnancy are likely to benefit from the knowledge that pregnancy and birth are generally normal and natural processes, their bodies are capable of growing and giving birth to a healthy baby, and complications are rare. Moreover, even when complications do occur, pregnancy care is designed to enable care providers to screen for and address such problems if they arise. Introducing young college-age women to the benefits of spontaneous vaginal birth with no or a minimum of interventions, and to the potential harms as well as the benefits of routine use of technological and pharmacological interventions is also likely to improve their capacity for effective decision making, and for feelings of control, when they do eventually become pregnant.

## Abbreviations

CS: Cesarean section
OECD: Organisation for Economic Co-operation and Development
